# The miRNAome of the postpartum dairy cow liver in negative energy balance

**DOI:** 10.1186/1471-2164-15-279

**Published:** 2014-04-12

**Authors:** Attia Fatima, David J Lynn, Padraic O’Boyle, Cathal Seoighe, Dermot Morris

**Affiliations:** 1 Animal and Bioscience Research Department, Animal & Grassland Research and Innovation Centre, Teagasc, Mellows Campus, Athenry, Co, Galway, Ireland; 2 School of Mathematics, Statistics and Applied Mathematics National University of Ireland Galway, Galway, Ireland; 3 Animal and Bioscience Research Department, Animal & Grassland Research and Innovation Centre, Grange, Dunsany, Co, Meath, Ireland

**Keywords:** micro RNA, RNA-seq, Liver, Negative energy balance, Nutrition, Dairy cattle

## Abstract

**Background:**

Negative energy balance (NEB) is an altered metabolic state in high yielding cows that occurs during the first few weeks postpartum when energy demands for lactation and maintenance exceed the energy supply from dietary intake. NEB can, in turn, lead to metabolic disorders and to reduced fertility. Alterations in the expression of more than 700 hepatic genes have previously been reported in a study of NEB in postpartum dairy cows. miRNAs (microRNA) are known to mediate many alterations in gene expression post transcriptionally. To study the hepatic miRNA content of postpartum dairy cows, including their overall abundance and differential expression, in mild NEB (MNEB) and severe NEB (SNEB), short read RNA sequencing was carried out. To identify putative targets of differentially expressed miRNAs among differentially expressed hepatic genes reported previously in dairy cows in SNEB computational target identification was employed.

**Results:**

Our results indicate that the dairy cow liver expresses 53 miRNAs at a lower threshold of 10 reads per million. Of these, 10 miRNAs accounted for greater than 95% of the miRNAome (miRNA content). Of the highly expressed miRNAs, miR-122 constitutes 75% followed by miR-192 and miR-3596. Five out of thirteen let-7 miRNA family members are also among the highly expressed miRNAs. miR-143, down-regulated in SNEB, was found to have 4 putative up-regulated gene targets associated with SNEB including *LRP2* (low density lipoprotein receptor-related protein 2), involved in lipid metabolism and up-regulated in SNEB.

**Conclusions:**

This is the first liver miRNA-seq profiling study of moderate yielding dairy cows in the early postpartum period. Tissue specific miR-122 and liver enriched miR-192 are two of the most abundant miRNAs in the postpartum dairy cow liver. miR-143 is significantly down-regulated in SNEB and putative targets of miRNA-143 which are up-regulated in SNEB, include a gene involved in lipid metabolism.

## Background

### miRNAs

miRNAs are among the most abundant and extensively studied class of small non-coding RNA. miRNAs are estimated to regulate the expression of up to 60% of mammalian protein coding genes [1] and are associated with many economically important traits in domestic livestock [2]. The biosynthesis of miRNA is comprised of multiple stages. The initial stage involves transcription of the miRNA gene to produce primary miRNAs, several hundred base-pairs in length, followed by the generation of 70 nucleotide-long hairpin shaped pre-miRNAs. Finally, Dicer-mediated processing results in the mature ~22 bp miRNA [3]. miRNA-mediated interference involves imperfect base-pairing of miRNAs, typically to the 3' UTR of the target mRNA. miRNAs have been reported to regulate gene expression in various biological pathways including those involved in metabolism and energy homeostasis [4]. In addition, miRNAs have been implicated in a wide variety of diseases [5,6]. The regulatory role of miRNAs in liver disease such as hepatitis, diet induced non-alcoholic fatty liver, alcoholic and non-alcoholic hepatocellular carcinoma and cirrhosis have been reported in humans and demonstrated in mouse liver studies [7-9]. In the case of beef cows, both liver specific and ubiquitously expressed miRNAs have been reported [10,11]. In another cow liver study, miRNAs were suggested as biomarkers for anabolic steroid abuse screening [12]. In addition, alterations in hepatic miRNAs in lambs have been associated with periconceptional changes in maternal nutrition [13].

### Negative energy balance

The postpartum period is of critical importance for the general and reproductive health of dairy cows [14]. During the early postpartum period energy expenditure for maintenance and lactation exceeds energy intake from nutrition resulting in NEB [15]. NEB increases the risk of other metabolic disorders such as ketosis and fatty liver and animals are prone to infectious diseases, including mastitis, due to reduced immunity [16]. NEB has profound effects on the liver, which undergoes many physiological and biochemical changes to counteract the energy imbalance [15,17]. An energy balance model of early lactation dairy cows was developed previously [18,19] resulting in two groups of cows with MNEB and SNEB. Alterations in gene expression in the liver associated with SNEB have been reported in two previous studies [19,20].

In the first study, 416 genes were found to be differentially expressed due to SNEB using microarray-based expression profiling [19]. In the second study, using RNA-seq, 413 genes were shown to be differentially expressed [20] with 72 differentially expressed genes in common between the two studies. Both of these studies report the alteration of genes involved in lipid metabolism the main biological process altered during SNEB. The mechanisms, however, mediating these alterations in transciptome expression are yet to be fully understood. In a microarray-based and RT-qPCR validated study of liver miRNAs from this NEB model, five miRNAs were found to be up-regulated in SNEB [21]. Microarray expression profiling, however, is limited to the pre-defined probe sets on the array and only relative expression of miRNAs can be measured. On the other hand with RNA sequencing (RNA-seq) it is possible to measure the expression of all putative miRNAs in a sample [22]. As miRNA activity and function depend on abundance [23], accurate quantification of miRNA abundance is important [24,25]. miRNA sequencing is based on short read RNA-seq and involves generation and high-throughput sequencing of 50 bp cDNA reads derived from the small RNA fraction of total RNA [26].

The objectives of this study were to (i) carry out an in-depth global analysis of the hepatic miRNA content of postpartum dairy cows, including their overall abundance and differential expression, in MNEB and SNEB using RNA sequencing and (ii) to computationally identify putative targets of differentially expressed miRNAs among differentially expressed hepatic genes reported previously in dairy cows in SNEB.

## Results

In total, more than 300 million reads were sequenced from eight cDNA libraries. Of these, more than 292 million reads passed quality control filters and just over 200 million reads aligned uniquely to Ensembl annotated genes in the UMD_3.1 assembly of the bovine genome. A summary of the data is provided in Table 1 and detailed statistics in Additional file 1: Table S1. 99% of reads, which aligned uniquely to the genome, aligned to known miRNAs (Additional file 2: Table S2). The UMD_3.1 build of the bovine genome has 26,618 annotated genes and miRBase version 16 includes 792 *Bos taurus* miRNAs including both pre and mature miRNAs. There were 479 miRNAs for which at least one read was observed in each of the eight samples in our dataset. Out of these, 53 miRNAs were expressed above a mean threshold of 10 reads per million (RPM) across all eight samples.

**Table 1:** Read statistics for SNEB and MNEB postpartum dairy cow liver miRNA-seq data

Sample^*^	**Processed reads**	**Untrimmed reads**	**Reads after QC**	**Reads aligning to UMD3.1.68†**	**Reads aligning uniquely to UMD3.1.68**	**Reads aligning to genes**
7S	35,531,177	319,143	33,034,345	29,929,732	22,008,536	21,617,227
8S	42,701,190	212,634	42,060,747	39,510,950	28,788,096	28,526,343
9S	39,733,845	276,697	35,308,457	32,324,504	24,940,747	24,682,635
10S	37,834,521	326,905	34,658,341	31,140,804	22,754,746	22,477,046
2M	35,079,188	272,113	32,996,280	30,024,759	23,042,362	22,809,423
3M	44,401,783	289,778	43,107,430	40,269,999	30,931,375	30,757,376
4M	40,699,727	280,820	32,868,631	29,571,511	23,373,573	23,050,065
5M	38,751,887	198,345	38,079,095	36,361,987	34,268,099	33,963,761
Sum	314,733,318	2,176,435	292,113,326	269,134,246	210,107,534	207,883,876
Average	39,341,665	272,054	36,514,166	33,641,781	26,263,442	25,985,485

*S = SNEB; M=MNEB.

†UMD3.1.68 = Ensembl v68 annotation of the UMD3.1 assembly of the bovine genome.

**Figure 1:**
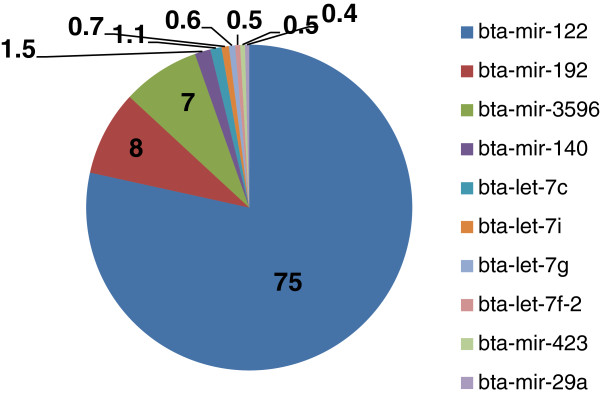
The 10 most abundant hepatic miRNAs in the postpartum dairy cow.

The 10 most highly expressed miRNAs accounted for more than 95% of all miRNAs expressed (Figure 1 and Table 2). The liver miRNA expression profile was dominated by miR-122, which accounted for 75% of all highly expressed miRNAs. This was followed by miR-192 which accounted for 8% and the let 7 family member miR-3596 which accounted for over 7% of the most abundantly expressed miRNAs. The remaining seven most highly abundant miRNAs were four members of the let 7 family (let-7c, let-7i, let-7 g and let-7f-2) which accounted for 3% while miR-140 accounted for ~1.5% and both miR-29a and miR-423 made up ~0.5% each (Figure 1). The most highly expressed liver miRNAs are all evolutionary conserved and include five members of the 13-member let-7 family (let-7c, let-7i, let-7 g, let-7f-2 and miR-3596). These miRNAs were located across nine different chromosomes with both let7-i and miR-3596 located on chromosome five. There was no evidence of clustering of highly expressed miRNAs in a particular genomic region. All are mature RNAs except for let-7f-2 a stem-loop miRNA. The relative abundances, sequences, genomic coordinates of highly expressed miRNAs are given in Table 2. One miRNA, miR-143 was differentially expressed between the SNEB and MNEB groups. miR-143 was 3.2-fold down-regulated in the SNEB group (FDR < 0.005) and was the 11^th^ most abundantly expressed (greater than 100,000 counts) miRNA The direction and magnitude of miR-143 regulation was confirmed (2.4-fold down-regulated) following RT-qPCR validation (Table 3).

**Table 2:** Highly expressed miRNAs in postpartum dairy cows

**miRNAs**	**miR family**	**miRNA-seq counts**	**Sequence**	**Coordinates (UMD3.1.68)†**
miR-122	miR-122	19364782	UGGAGUGUGACAAUGGUGUUUG	chr24: 58095642–58095726 [+]
miR-192	miR-192	2083071	CUGACCUAUGAAUUGACAGCCAG	chr29: 43731660–43731765 [+]
miR-3596	let 7	1902520	AACCACACAACCUACUACCUCA	chr5: 117120188–117120270 [−]
miR-140	miR-140	385996	UACCACAGGGUAGAACCACGGA	chr18: 37088137–37088230 [+]
bta-let-7c	let 7	274368	UGAGGUAGUAGGUUGUAUGGUU	chr1: 19930459–19930542 [−]
bta-let-7i	let 7	179868	UGAGGUAGUAGUUUGUGCUGUU	chr5: 51209081–51209164 [−]
bta-let-7 g	let 7	150545	UGAGGUAGUAGUUUGUACAGUU	chr22: 49189340–49189422 [+]
bta-let-7f-2	let 7	124091	UGUGGGAUGAGGUAGUAGAUUGUAUAGUUUUAGGGUCAUACCCCAUCUUGGAGAUAACUAUACAGUCUACUGUCUUUCCCACG	chrX: 96383532–96383614 [−]
miR-423	miR-423	116540	AAGCUCGGUCUGAGGCCCCUCAGU	chr19: 21799484–21799577 [+]
miR-29a	miR-29	115428	CUAGCACCAUCUGAAAUCGGUUA	chr4: 95402319–95402382 [−]

†UMD3.1.68 = Ensembl v68 annotation of the UMD3.1assembly of the bovine genome.

**Table 3:** Differentially expressed miRNA in SNEB postpartum dairy cow liver validated using RT-qPCR

**Ensemble gene**	**miRBase ID**	**FC**	**FDR**
ENSBTAG00000030114	bta-mir-143	−2.40	0.029

Differential expression analysis was carried out with PROC t-test (SAS) after normalization of Ct-values to reference gene RNU6B.

In order to identify genes that may be affected by the down-regulation of miR-143 in SNEB, we computationally predicted miRNA targets among the 3’UTRs of a combined set of 757 hepatic genes that have been previously reported to be differentially expressed in the liver tissues of the same animals [19,20]. A total of 4 genes reported to be up-regulated in SNEB were found to be putative targets of miR-143 (Table 4). The biological functions and intracellular location of these targets are given in Table 5.

**Table 4:** **Up-regulated putative hepatic gene targets of miR-143 in postpartum dairy cows in SNEB**^**†**^

**Human gene name**	**Fold change**	**FDR**
*CHRM1*	4.3	0.004222
*LRP2*	3.28	0.015161
*ARHGEF40*	2.85	1.34E-06
*C6orf145*	2.1	1.02E-06

^**†**^From Mc Carthy et al., [19]; McCabe et al., [20].

**Table 5:** **Functional categories of up-regulated putative target hepatic genes of miR-143**^**†**^

**ID**	**Symbol**	**Entrez gene name**	**Location**	**Type(s)**
*CHRM1*	*CHRM1*	cholinergic receptor, muscarinic 1	Plasma Membrane	G-protein coupled receptor
*LRP2*	*LRP2*	low density lipoprotein receptor-related protein 2	Plasma Membrane	Transporter
*ARHGEF40*	*ARHGEF40*	Rho guanine nucleotide exchange factor (GEF) 40	unknown	Enzyme
*C6orf145*	*PXDC1*	PX domain containing 1	unknown	Signalling protein

^†^Target genes are those predicted using both TargetScan and miRanda.

## Discussion

The postpartum increase in milk production in high yielding dairy cows is accompanied by increased nutritional and energetic demands. This results in 10–12 weeks of NEB in all high-yielding dairy cows [26]. Previous reports have shown that SNEB affects the expression of multiple genes in the liver, including genes involved in lipid and glucose metabolism and homeostasis [19,20]. Hepatic miRNAs have been reported to play a role in hepatic functions and disorders in human and mouse [27]. In addition miRNAs are reported to be associated with energy metabolism through their role in modulation of glucose and lipid homeostasis [28,29]. The miRNA profile of MNEB and SNEB dairy cow liver two weeks postpartum in this study shows some degree of similarity with hepatic miRNA profiles of other animals including human, mouse, rat, and beef cows.

### Abundantly expressed miRNAs in postpartum dairy cow liver

This is the first liver miRNA-seq profiling study of moderate yielding dairy cows in the first two weeks postpartum. The most dominant among the ten highly abundant miRNAs in our study, miR-122, is a liver-specific conserved miRNA. This dominance of expression by miR-122 is consistent with previous studies in dairy [10] and beef cows [11] where miR-122 was reported to be only expressed in liver, when compared with other tissues, and constituted more than 57% of all the miRNA reported in liver [11]. In addition, miR-122 has been reported to account for 70% of the total miRNAs in human liver [30]. Tissue specific roles of miR-122 are well established in human and miR-122 has been implicated in the hepatic disorders hepatocarcinogenesis and hepatocellular carcinoma [9,31,32]. Furthermore, the antagonism of miR-122 was reported to result in disruptions of cholesterol and lipid metabolism in mice [9]. The second most abundant miRNA in our study miR-192 has been associated with cellular responses to glucose stimulus [33] and was also reported to be one of the most highly expressed after miR-122 in human liver miRNA studies [31,34] and in a mouse liver study [35]. miR-192 unlike miR-122 is not a liver specific miRNA and has also been reported to have important roles in human and bovine renal tissues and functions [36-38]. Five of the thirteen members of the let-7 family are also highly expressed in this study including let-7c, let-7i, let-7 g, miR-3596 and let-7-f. The let-7 family members have been associated with hepatic development and disorders as well as glucose and insulin metabolism [39]. Over-expression of let-7c in human liver was implicated in hepatocytes oxidant injury [40] while let-7 g was demonstrated to suppress HCC metastasis in a mouse model [41]. miR-140, which is also among the highly expressed miRNAs in this study, has been reported to be abundantly expressed in human and mouse liver with implications in liver function and disorders [42-44].

### Differentially expressed miRNAs in postpartum dairy cow liver in SNEB

Only one miRNA, miR-143 was found to be differentially expressed in this study using miRNA-seq and subsequently validated by RT-qPCR. This may have been due in part to the fact that most of the sequence reads in this study came from a small number of miRNAs possibly limiting the power of RNA-seq to accurately estimate the abundance of the more lowly expressed miRNAs. Less abundant miRNAs would not be affected to the same extent in a microarray based approach which has probes corresponding to known miRNAs. In a previous microarray-based study of the same animals, ten miRNAs were found to be differentially expressed; five of which were validated using RT-qPCR [21]. In this study, one additional miRNA bta-miR-1247, would have been declared differentially expressed were it not excluded from the analysis due to having read counts below the lower threshold criterion of 10 reads per million. In addition, in this study a cut-off of 18 nts in length was used in the analysis, whereas in the microarray study, miR-1281 which was found to be differentially expressed is 17 nts in length.

miR-143, a highly expressed miRNA in this study, is down-regulated in SNEB. miR-143 is not liver-specific, however its tissue-enriched expression has been reported in human [45] chicken [46], rat [43] and mouse [47,48] liver. In the case of human, miR-143 has been implicated in hepatocellular carcinoma. In the rat study, miR-143 was demonstrated to regulate activation and proliferation of myofibroblastic hepatic stellate cells (HSC) while in the mouse study miR-143 was shown to regulate glucose metabolism *in vitro*, and was associated with hepatic insulin regulation. In addition, miR-143 has been found to be ubiquitously expressed in bovine tissues including liver [10] and has also been associated with bovine intramuscular fat proliferation and differentiation [49] and high back fat deposition in crossbred beef cattle [50].

### Some important predicted gene targets of miRNA miR-143

Of the 4 putative targets of miR-143 which have been reported previously to be up-regulated in SNEB, *LRP2* is involved in lipid metabolism and is of particular interest because the metabolism of lipids is altered during SNEB [51,52]. *LRP2* is implicated in lipid metabolism through its role as a receptor for sterols, steroid hormones bound to carrier proteins [53] like lipoproteins [54] and apolipoprotein M of liver [55-57]. A study of miRNA based modulation of obesity also reported *LRP2* as a putative target of miR-130a, with roles in lipid metabolism [58].

## Conclusions

From a global examination of miRNA expression in the liver of postpartum dairy cattle ten highly expressed miRNAs dominate. Tissue specific miR-122 and liver enriched miR-192 are two of the most abundant miRNAs in the postpartum dairy cow liver. miR-143 is significantly down-regulated in SNEB and putative targets of miRNA-143 up-regulated in SNEB, include *LRP2* a good candidate as a potential mediator of the metabolic effects of NEB.

## Methods

### NEB model

A NEB dairy cow model developed previously was used. In this model differential feeding and milking regimes were used to produce two groups of Holstein Friesian cows; MNEB and SNEB [18,19]. Briefly, MNEB cows were fed *ad libitum* grass silage with 8 kg/day of a 21% crude protein dairy concentrate and milked once daily. SNEB cows were fed 25 kg/day silage with 4 kg/day concentrate and milked thrice daily.

### Liver tissue collection for miRNA analysis

All procedures were carried out under license in accordance with the European Community Directive, 86-609-EC. Cows were slaughtered approximately 14 days postpartum (MNEB; 13.6 ± 0.75, range 11–15; SNEB 14.3 ± 0.56, range 13–16) and the entire liver was removed within 15 to 30 min [19]. Samples weighing approximately 1 g were dissected, rinsed in RNAase-free phosphate buffer, snap-frozen in liquid nitrogen and stored at -80°C. Liver tissue samples from 4 animals from each group were used for miRNA library preparation.

### RNA extraction and library preparation

Total RNA enriched for small RNA was extracted from 1 mg of frozen tissue from each of the eight samples using the mirVana™ miRNA Isolation Kit (Life Technologies, Carlsbad, CA, USA) according to the manufacturer’s protocol. The RNA extract was stored at −80°C. An Agilent RNA 6000 Nano Kit and the 2100 Bioanalyzer was used to measure total RNA integrity (Agilent Technologies, Colorado Springs, CO, USA) and the Agilent Small RNA Kit (Agilent Technologies) was used for miRNA quantification. Small RNA libraries were constructed at BGI (Shenzhen, China) under contract and sequenced on an Illumina HiSeq2000 platform.

### Data analysis pipeline

FASTQC v0.10.0 (http://www.bioinformatics.babraham.ac.uk/projects/fastqc/) was used to carry out preliminary quality control of the 8 FASTQ files. The 3′ adaptor sequences were trimmed with Cutadapt v1.1 (https://code.google.com/p/cutadapt). After trimming, reads shorter than 18 nucleotides were discarded. The FASTQ quality filter v0.0.13 (http://hannonlab.cshl.edu/fastx_toolkit/) was applied to trim low quality bases from reads. Phred scores were calculated and reads where at least 50% of the bases had a Phred score of less than 20 were discarded [59]. The filtered reads were further trimmed at their ends to remove low quality bases (Phred score <20). Reads were then aligned to the bovine genome (UMD3.1) using Novoalign version 2.07.11 (http://www.novocraft.com) using the “-m” miRNA mode. Only uniquely aligned reads were retained. HTSeq version 0.5.3p3 (http://www-huber.embl.de/users/anders/HTSeq/doc/overview.html) was used to annotate uniquely aligned reads using Ensembl (v68) bovine gene and miRNA annotation.

### Differential expression analysis

As differential expression analysis of miRNAseq data is sensitive to the normalisation method a number of alternative methods were used [60]. Differential expression was assessed following normalisation of count data using either the trimmed mean of M-values (TMM), the upper quartile-normalisation method or no normalisation using the Bioconductor package EdgeR (v2.4.6). EdgeR which uses a negative binomial model to account for both biological and technical variability was used to identify differentially expressed miRNAs between the two groups using moderated tag wise dispersions. A Benjamini and Hochberg [61] corrected P value cut-off of <0.05 was applied to correct for multiple testing. Only miRNAs expressed above a threshold of 10 reads per million were considered for the differential expression analysis as miRNAs expressed below this threshold are unlikely to be functional.

### RT-qPCR validation of differentially expressed miRNAs

RT-qPCR was used for technical validation of differentially expressed miRNA using TaqMan miRNA assay (Applied Biosystems, Dublin, Ireland). Gene-specific reverse transcription was performed on 10 ng of purified total RNA using the TaqMan MicroRNA Reverse Transcription kit according to manufacturer’s instructions (Applied Biosystems, Dublin, Ireland). RT-qPCR reactions were performed using 1 μl of cDNA (10 ng/μl) in 9 μl of Taqman universal master mix containing TaqMan PCR primers and probes on a BioRad CFX96 real time PCR system (Bio-Rad, United Kingdom) using the following cycling parameters: 95°C for 10 min followed by 40 cycles at 95°C for 15 s and 60°C for 1 min. A total of four biological replicates from each of the MNEB and SNEB treatment groups were used for RT-qPCR validation. From prior experience with microarray hepatic miRNA expression validation and using the same liver tissue samples from the same animals, *RNU6B* was chosen as the most stable internal reference miRNA [21]. miRNA expression levels were recorded as Ct values, i.e., the number of PCR cycles at which the fluorescence signal is detected above the threshold value. The software package BioRad CFX manager was used for correction of the Ct values and normalization to RNU6B using the 2^-ΔΔCt^ method [62]. Corrected Ct values were used to calculate differential expression using the PROC t-test (SAS) [63]. A P < 0.05 was deemed to be significant.

### MiRNA target predictions

Both TargetScan Release 6.2 (http://www.targetscan.org) [1,64,65] and miRanda v4.0 (http://www.microrna.org) [66] were used to predict common targets of differentially expressed miRNAs among the two datasets of up-regulated hepatic genes previously reported for liver tissues from the same cows [19,20].

## Availability of supporting data

The data sets supporting the results of this article are available in Gene Expression Omnibus (GEO) repository GSE55882 http://www.ncbi.nlm.nih.gov/geo/query/acc.cgi?acc=GSE55882.

## Competing interests

The authors declare that they have no competing interests.

## Authors’ contributions

DM, CS conceived and designed the experiments. AF, PO performed the experiments. AF, CS, DL, DM analysed the data. AF, CS, DM, DL drafted the manuscript. All authors read and approved the final manuscript.

## Authors’ information

Cathal Seoighe and Dermot G Morris are joint senior authors.

## Supplementary Material

Additional file 1: Table S1 Detailed summary of RNA-seq data for postpartum dairy cow liver. Click here for file

Additional file 2: Table S2 Percentage distribution of RNA biotypes. Click here for file
