# Supplementary material for: The miRNAome of the postpartum dairy cow liver in negative energy balance
Source: BMC Genomics. 2014 Apr 12;15:279. doi: 10.1186/1471-2164-15-279 (PMC4023597; doi:10.1186/1471-2164-15-279)
Supplement: Additional file 1: Table S1 — Detailed summary of RNA-seq data for postpartum dairy cow liver. [file 1471-2164-15-279-S1.doc]

**Supplementary Table 1.** Detailed summary of RNA-seq data for postpartum dairy cow liver.

| **Sample** | **Processed Reads** | **Untrimmed Reads** | **Too Short Reads** | **Reads for QC** | **Reads after QC** | **Reads Aligning to UMD3.1.68** | **Reads Aligning Uniquely to UMD3.1.68** | **Reads Aligning to Genes** |
| --- | --- | --- | --- | --- | --- | --- | --- | --- |
| 7S | 35,531,177 | 319,143 | 811,013 | 34,401,021 | 33,034,345 | 29,929,732 | 22,008,536 | 21,617,227 |
| 8S | 42,701,190 | 212,634 | 297,204 | 42,191,352 | 42,060,747 | 39,510,950 | 28,788,096 | 28,526,343 |
| 9S | 39,733,845 | 276,697 | 3,830,181 | 35,626,967 | 35,308,457 | 32,324,504 | 24,940,747 | 24,682,635 |
| 10S | 37,834,521 | 326,905 | 1,374,303 | 36,133,313 | 34,658,341 | 31,140,804 | 22,754,746 | 22,477,046 |
| 2M | 35,079,188 | 272,113 | 464,663 | 34,342,412 | 32,996,280 | 30,024,759 | 23,042,362 | 22,809,423 |
| 3M | 44,401,783 | 289,778 | 649,851 | 43,462,154 | 43,107,430 | 40,269,999 | 30,931,375 | 30,757,376 |
| 4M | 40,699,727 | 280,820 | 7,272,269 | 33,146,638 | 32,868,631 | 29,571,511 | 23,373,573 | 23,050,065 |
| 5M | 38,751,887 | 198,345 | 414,804 | 38,138,738 | 38,079,095 | 36,361,987 | 34,268,099 | 33,963,761 |
| Sum | 314,733,318 | 2,176,435 | 15,114,288 | 297,442,595 | 292,113,326 | 269,134,246 | 210,107,534 | 207,883,876 |
| Average | 39,341,665 | 272,054 | 1,889,286 | 37,180,324 | 36,514,166 | 33,641,781 | 26,263,442 | 25,985,485 |
