# Supplementary material for: The miRNAome of the postpartum dairy cow liver in negative energy balance
Source: BMC Genomics. 2014 Apr 12;15:279. doi: 10.1186/1471-2164-15-279 (PMC4023597; doi:10.1186/1471-2164-15-279)
Supplement: Additional file 2: Table S2 — Percentage distribution of RNA biotypes. [file 1471-2164-15-279-S2.doc]

**Additional file 2: Table S2.** Percentage distribution of RNA biotypes

| **Gene Biotype** | **Percentage** |
| --- | --- |
| miRNA | 99.06 |
| misc_RNA | 0.02 |
| mt_rRNA | 0.08 |
| mt_tRNA | 0.04 |
| protein_coding | 0.59 |
| pseudogene | 0.001 |
| retrotransposed | 0.0003 |
| rRNA | 0.00036 |
| snoRNA | 0.198 |
| snRNA | 0.0012 |
